# Suv4-20h Abrogation Enhances Telomere Elongation during Reprogramming and Confers a Higher Tumorigenic Potential to iPS Cells

**DOI:** 10.1371/journal.pone.0025680

**Published:** 2011-10-12

**Authors:** Rosa M. Marión, Gunnar Schotta, Sagrario Ortega, Maria A. Blasco

**Affiliations:** 1 Telomeres and Telomerase Group, Molecular Oncology Program, Spanish National Cancer Centre, Melchor Fernández Almagro 3, Madrid, Spain; 2 Munich Center for Integrated Protein Science and Adolf-Butenandt-Institute, Ludwig-Maximilians-University, Munich, Germany; 3 Transgenic Mice Unit, Biotechnology Program, Spanish National Cancer Centre, Melchor Fernández Almagro 3, Madrid, Spain; St Jude Children's Research Hospital, United States of America

## Abstract

Reprogramming of adult differentiated cells to induced pluripotent stem cells (iPS) cells has been achieved by over-expression of specific transcription factors. Nuclear reprogramming induces a series of profound changes at the telomeres of the parental differentiated cells, including a telomerase-dependent telomere elongation and the remodeling of telomeric chromatin. In particular, iPS cells show a decreased density of H4K20me3 heterochromatic mark at telomeres compared to the parental cells. Suv4-20h1 and Suv4-20h2 histone methytransferases (HMTases) are responsible for the trimethylation of H4K20 at telomeres, as cells deficient for both HMTases show decreased levels of H4K20me3 at telomeric chromatin. Here, we set to address the role of the Suv4-20h enzymes in telomere reprogramming by generating bona-fide iPS cells from mouse embryonic fibroblasts (MEFs) double null for both HMTases (*Suv4-20dn* MEFs). We found that Suv4-20h deficiency enhances telomere elongation during reprogramming without altering their ability to protect the chromosome ends or the efficiency of reprogramming. Moreover, teratomas generated from *Suv4-20dn* iPS cells also have elongated telomeres and an increased growth rate when compared to wild-type controls. These results indicate that abrogation of Suv4-20h enzymes and loss of heterochromatic mark H4K20me3 at telomeric heterochromatin facilitates telomere reprogramming and provides an increased tumorigenic potential to the resulting iPS cells.

## Introduction

Mouse and human somatic cells can be reprogrammed to the so-called induced pluripotent stem (iPS) cells by simultaneous over expression of four or less transcription factors related to stem cell pluripotency [1], [2], [3], [4], [5], [6]. These cells have enormous potential for the generation of patient-specific cells to be used for regenerative medicine, *in vitro* modeling of human diseases, and drug discovery. The molecular mechanisms by which the transcription factors enable this de-differentiation process are not fully understood, but *in vitro* reprogramming involves the acquisition of an embryonic stem cell gene expression profile and global epigenetic changes [5], [6], [7], [8]. Thus, epigenetic marks are properly reprogrammed during iPS cell generation, reaching a pattern that resembles that of ES cells, with a more open chromatin compared to differentiated cells [9]. The remodeling of the chromatin to a more relaxed conformation by the removal of the multilayered marks of epigenetic silencing, such as histone and DNA methylation, constitute an essential part of the de-differentiation process. In accordance, several chromatin-remodeling proteins, as well as demethylation-promoting agents and histone deacetylase inhibitors that promote chromatin opening have been shown to regulate reprogramming [8], [10], [11], [12], [13].

Telomeres are reprogrammed during mouse iPS cell generation to adopt features similar to those characteristic of ES cell telomeres [14], [15]. Telomeres are heterochromatic structures at the end of chromosomes that protect them from degradation and from being detected as double-strand DNA breaks [16], [17]. Telomeres comprise complexes of tandem DNA repeats bound by a specialized multiprotein complex known as shelterin [17]. Mammalian telomere length and integrity play an important role in processes such cancer and aging, characterized by defects in telomere length [18]. Thus, telomeres have been shown to shorten associated to increasing age [19] and contribute to organismal aging by limiting the proliferative capacity of adult stem cells [20], [21], [22]. Telomere length is maintained by telomerase, a reverse transcriptase enzyme [23] whose expression is restricted to embryonic development as well as to adult stem cell compartments [20], [21], [22], [24]. Telomere-elongation is in turn regulated by the epigenetic status of telomeric chromatin [25], [26]. In particular, telomeric and subtelomeric regions are enriched in histone marks characteristic of repressed heterochromatin domains, such as trimethylation of H3K9 and H4K20 and binding of heterochromatin protein 1 (HP1) [27], [28], [29] and subtelomeric DNA is heavily methylated [29]. Loss of these heterochromatic marks is concomitant with excessive telomere elongation [27], [28], [29].

During reprogramming, a telomerase-dependent telomere elongation occurs in iPS cells derived from mouse embryonic fibroblasts (MEFs), which continue post-reprogramming until reaching ES cell telomere length [15]. Moreover, generation of iPS cells involves a change in the epigenetic status of telomeres, demonstrating that telomeric chromatin is dynamic and reprogrammable depending of the differentiation stage of cells. In particular, iPS cells show a decreased density of H4K20me3 at telomeric repeats [14], [15] compared to the parental cells. It is thought that chromatin remodeling is a requisite for telomerase-dependent telomere elongation during iPS cell generation.

In has been described that the enzymatic activities responsible for the trimethylation of H4K20 at telomeres are the Suv4-20h histone methytransferases [27]. Murine Suv4-20h1 and Suv4-20h2 are involved in di- and trimethylation of H4K20 in heterochromatic domains, where they play an important role in chromatin maintenance [27], [30], [31]. Cells simultaneously deficient for both enzymes, Suv4-20 double-null (dn), show a chromatin-wide transition to H4K20 monomethylation and a marked decrease in H4K20me3 at pericentric and telomeric chromatin compared to wild-type controls [27], [31], [32]. In view of the role of Suv4-20h enzymes in maintenance of the H4K20me3 heterochromatic mark on telomeric chromatin and the loss of this mark on telomeres during iPS cell generation, we asked whether Suv4-20h HMTases may play a role in regulating telomere reprogramming. Here, we show that abrogation of Suv4-20h enzymes accelerates telomere elongation during reprogramming. Also, *Suv4-20dn* iPS cells-induced teratomas show an increased growth rate and elongated telomeres when compared to wild-type controls. Our results indicate that loss of heterochromatic mark H4K20me3 in telomeric heterochromatin facilitates telomere reprogramming.

## Materials and Methods

### Cell culture

Primary MEFs derived from wild type or Suv4-20dn embryos (C57BL6 genetic background) were a kind gift from Thomas Jenuwein. MEF were cultured in standard DMEM medium with 10% FBS (Gibco). iPS cells were cultured in DMEM (high glucose) supplemented with serum replacement (KSR, Invitrogen), LIF 1000 u/ml, non-essential amino acids, glutamax and beta-mercaptoethanol.

### Generation of mouse iPS cells

Reprogramming of primary (passage 2–4) mouse embryo fibroblasts derived either from wild type or Suv4-20dn embryos (MEFs, of C57BL6 genetic background) was performed essentially as previously described by us [15] following modifications of a previous protocol [33]. Briefly, retroviral supernatants were produced in HEK-293T cells (ATCC® Number CRL-11268™) (5×10^6^ cells per 100-mm-diameter dish) transfected with the ecotropic packaging plasmid pCL-Eco (4 µg) together with either one of the following retroviral constructs (4 µg), pMXs-Klf4, pMXs-Sox2 or pMXs-Oct3/4 (obtained from Addgene). Transfections were performed using Fugene-6 transfection reagent (Roche) according to the manufacturer's protocol. Two days later, retroviral supernatants (10 ml) were collected serially during the subsequent 48 hours, at 12 hour intervals, each time adding fresh medium to the cells (10 ml). The recipient MEFs had been seeded the previous day (2×10^5^ cells per 60-mm-diameter dish) and received 1 ml of each of the corresponding retroviral supernatants (a total of three). This procedure was repeated every 12 hours for 2 days (a total of 4 additions). After infection was completed, media was replaced with standard ES media supplemented with knockout serum replacement (KSR, Invitrogen). Cultures were maintained in the absence of drug selection with daily medium changes. Reprogramming was assessed 2 weeks post-infection by counting alkaline phosphatase-positive colonies. Alkaline phosphatase staining was performed according to manufacturer's instructions (Alkaline Phosphatase Detection kit (Millipore). The results were normalized to the respective efficiencies of retroviral transduction as assessed by transducing with the three pMXsOct3/4, pMXsKlf4, pMXsSox2 retroviruses plus a retrovirus expressing GFP. Colonies were picked after 2 weeks and expanded on feeder fibroblasts using standard procedures.

### iPS-chimeras

The capacity of the *wt* and *Suv4-20dn* iPS clones to generate chimeras *in vivo* was tested by microinjection into C57BL/6J-*Tyr*(C-2J)/J (albino) blastocysts, or by aggregation with CD1 (albino) morulae and assessment of hair color in the resulting progeny. Mice were treated in accordance with the Spanish Laws and the Guidelines for Humane Endpoints for Animals Used in Biomedical Research. The Spanish National Cancer Research Centre (CNIO) is part of the “Carlos III” Health Institute (ISCIII) and all protocols were previously subjected and approved by the Ethical Committee of the ISCIII; approval ID numbers: PA-311, PA- 352.2 and PA-142/07.

### Teratoma formation

Mice (nu/nu) were subcutaneously injected with 1×10^6^ cells of each iPS cell clone. Tumor growth was measured at the indicated days post-injection with a caliber, and tumor volume was calculated according to the formula: long diameter×(short diameter)^2^×0.51.

### Histopathology and immunohistochemistry

After mice excision, the teratomas were fixed in 10% buffered formalin (Sigma) and embedded in paraffin. For histopathological analysis of teratomas, tumours were sectioned and sections were stained with haematoxylin and eosin according to standard procedures. For immunohistochemical studies Nanog antibody (Novus Biologicals, NB100-58842, 1∶800) and Oct3/4(H-134) antibody (Santa Cruz Biotechnology, sc-9081, 1∶150) were used following manufacturer's instructions. Following incubation with the primary antibodies, positive cells were visualized using 3,3-diaminobenzidine tetrahydrochloride plus (DAB^+^) as a chromogen.

### Western blot

Cell extracts were prepared using RIPA buffer, resolved on NuPAGE 4–12% gradient Bis-Tris gels, transferred to nitrocellulose and hybridized using Abs against Nanog (1∶1000; Chemicon AB 5731), Oct4 (H-134) (1∶500; SantaCruz sc-9081) and tubulin (1∶10000 SIGMA T6557).

### TRF analysis

Cells were included in agarose plugs, and TRF analysis was performed as described previously [34].

### Chromosomal aberrations

FISH hybridization was performed as described before [29], [35]. At least 12 metaphases per genotype were scored for chromosomal aberrations by superimposing the telomere image on the DAPI chromosomes image using the TFL-telo software.

### Telomere length analysis using telomere Q-FISH on metaphases

We prepared metaphases and performed Q-FISH hybridization as previously described [29], [35]. To correct for lamp intensity and alignment, images from fluorescent beads (Molecular Probes, Invitrogen) were analyzed in parallel, using the TFL-Telo program (a gift from P. Lansdorp, Terry Fox Laboratory, British Columbia Cancer Research Centre, Vancouver, Canada). Telomere fluorescence values were extrapolated from the telomere fluorescence of lymphoma cell lines LY-R (R cells) and LY-S (S cells) with known telomere lengths of 80 and 10 kb, respectively. There was a linear correlation (r^2^ = 0.999) between the fluorescence intensity of the R and S telomeres. We captured the images using a CCD camera (FK7512; COHU) on a fluorescence microscope (DMRB; Leica). We captured the images using Q-FISH software (Leica) in a linear acquisition mode to prevent the oversaturation of fluorescence intensity. TFL-Telo software [36] was used to quantify the fluorescence intensity of telomeres from at least 5–10 metaphases for each data point.

### Telomere length analysis of teratoma sections

Teratoma sections were deparaffinated and quantitative telomere fluorescence *in situ* hibridization (Q_FISH) was perfomed as described [37].

### ChIP assay

ChIP assays were performed as previously described [28]. In brief, after cross-linking and sonication, chromatin from 4×10^6^ cells were used per each immunoprecipitation with protein A/G Plus agarose beads (Santa Cruz Biotechnology, sc-2003) and the following antibodies: 6 ug of anti-histone H3 (# ab1791, Abcam), 6 ug of anti-H3K9me3 (#07-442, Upstate Biotechnology), 6 ug of anti-H4K20me3 (# 07-749, Upstate Biotechnology), 8 µl of rabbit polyclonal antibody to TRF1, raised in our laboratory against full-length mouse TRF1 protein, or preimmune serum. The immunoprecipitated DNA was transferred to a Hybond N^+^ membrane using a dot blot apparatus. The membrane was then hybridized with either a telomeric probe containing TTAGGG repeats or a probe recognizing major satellite sequences, which is characteristic of pericentric heterochromatin. Quantification of the signal was performed with ImageJ software. The amount of telomeric and pericentric DNA after ChIP was normalized to the total telomeric or centromeric DNA signal respectively for each genotype, as well as to the H3 abundance at these domains, thus correcting for differences in the number of telomere repeats or in nucleosome spacing.

### Telomere transcription

Telomere transcription was studied as described [38]. Total RNA was prepared using Trizol (Invitrogen). Northern blotting was performed as described previously [38]. The 1,6 kb telomere DNA probe used to detect the telomeric RNA was a kind donation from Dr. Titia de Lange, Rockefeller University, NY, USA. The DNA probe was labelled by random priming (Rediprime, GE healthcare).

## Results

### *Suv4-20dn* cells can be efficiently reprogrammed into *bona-fide* iPS cells

**Figure 1:**
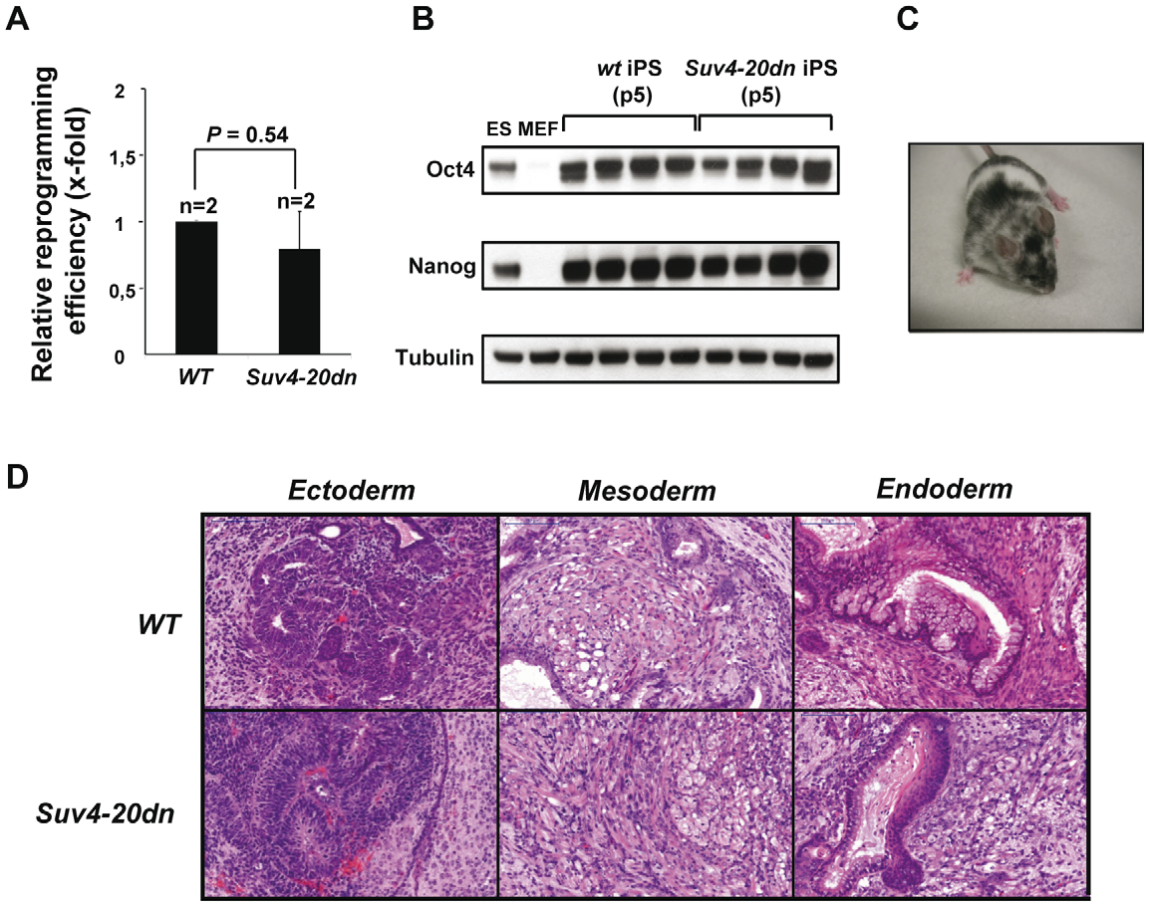
Generation of *bona-fide* pluripotent mouse Suv4-20dn iPS cells. **a.** Quantification of relative reprogramming efficiency obtained with *Suv4-20dn* MEF compared to that obtained with wild-type MEF. Efficiency of iPS generation was calculated as the number of alkaline phosphatase- positive colonies and normalized to retroviral infection efficiency as determined by GFP fluorescence (see Methods). Note that the efficiency of iPS generation is similar in *Suv4-20dn* MEF compared to the wild-type controls. Indicated statistics was performed using a Student's t-test. Error bars indicate the standard error. n, represents number of independent MEF cultures. **b.** Robust expression of endogenous pluripotency markers Oct4 and Nanog in *wt* and *Suv4-20dn* iPS cells (passage 5) as measured by *western-blot*. Four individual iPS cell clones per genotype are shown. Note that wild-type and *Suv4-20dn* iPS cells express similar levels of endogenous Nanog and Oct4 than ES cells of the same genetic background (C57BL6), while MEF do not express these factors. **c.** Representative example of *Suv4-20dn* iPS cells contribution to mouse chimerism. **d.** Representative examples of the teratomas generated with *wt* and *Suv4-20dn* iPS cells 24 days after injection. Note that teratomas from both genotypes show tissues derived from the three germ layers (ectoderm, mesoderm, endoderm). Scale bar, 100 µm.

*Wt* and *Suv4-20dn* mouse embryonic fibroblasts (MEFs) were reprogrammed using retroviral vectors expressing a combination of three reprogramming factors (Oct4, Klf4 and Sox2) previously shown by us to reprogram wild-type MEFs into *bona fide* pluripotent iPS cells [15]. Reprogramming efficiency was calculated as the number of iPS cell colonies obtained at day 14 post-infection with the three reprogramming factors relative to the total number of cells initially infected (infection efficiency was measured in quadruple infections with the three factors plus GFP, all at equal proportions, and analyzed by flow cytometry to detect the proportion of GFP-positive cells). *Suv4-20dn* MEFs were reprogrammed with similar efficiency to that of *wt* MEFs (Fig. 1A), indicating that loss of H4K20 trimethylation in heterochromatic regions does not alter the reprogramming capacity of the cells. The resulting iPS cell colonies we isolated, expanded and tested for pluripotency by several means. First, both *wt* and *Suv4-20dn* iPS cells showed a robust expression of the pluripotency markers Oct4 and Nanog, as measured by *Western-blot* (Fig. 1B). Next, we confirmed their ability to contribute to mouse chimerism when microinjected into C57BL6-Tyr^c^ (albino) blastocysts or when aggregated with CD1 (albino) morulae (Fig. 1C and Table S1). Finally, both *wt* and *Suv4-20dn* iPS cells were able to induce teratomas when injected in immunodeficient mice, with presence of tissues derived from all the three germ layers (Fig. 1D) and with similar levels of cell differentiation as measured by Oct4 and Nanog staining (Fig. S1). Altogether, these results indicate that MEFs lacking the HMTases Suv4-20h1 and Suv4-20h2 activities can be efficiently reprogrammed into *bona-fide* iPS cells.

### Reprogramming of telomeric chromatin in *Suv4-20dn* iPS cells

**Figure 2:**
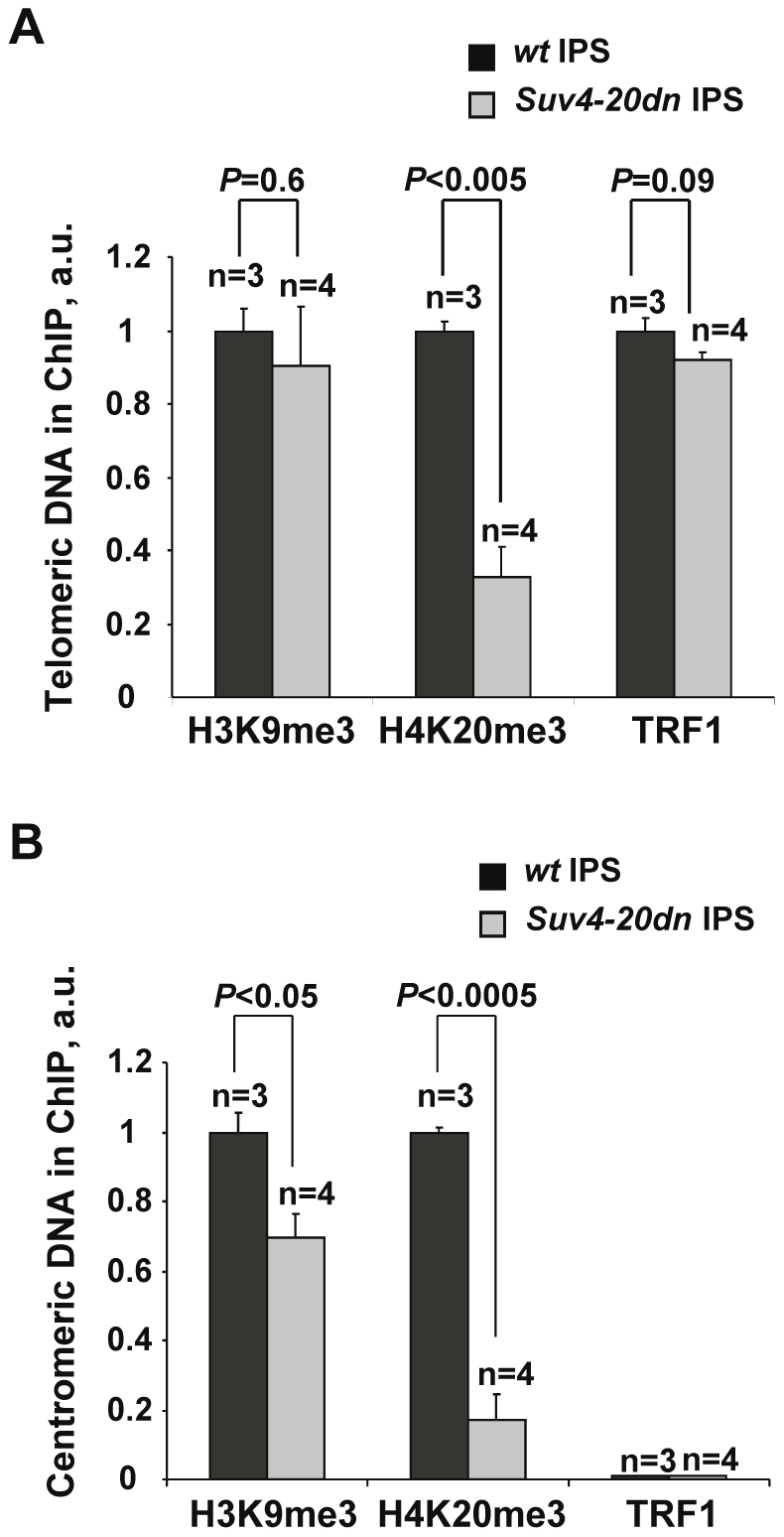
Reprogramming of telomeric chromatin in *Suv4-20dn* iPS cells. **a–b.** ChIP of *wt* and *Suv4-20dn* iPS cells (passage 9) with the indicated antibodies. Quantification of immunoprecipitated telomeric (**a**) and pericentric (**b**) repeats was normalized to input signals. ChIP values are represented as percentages of the wild-type, which was set to 1. Note that H4K20me3 mark is drastically reduced in *Suv4-20dn iPS* cells when compared to *wt* iPS cells, while H3K9me3 and TRF1 levels at telomeres are similar in both genotypes. n represents number of independent iPS cells clones. Indicated statistics was performed using a Student's t-test. Error bars correspond to standard error.

Along the reprogramming process the epigenetic signature of telomeric heterochromatin is changed, and the density of H4K20me3 heterochromatic mark is decreased compared to differentiated MEFs. [15]. We previously reported [27] that *Suv4-20dn* MEFs show a lower density of H4K20me3 at telomeric chromatin compared with wild-type MEFs, demonstrating that the Suv4-20h HMTases are responsible for the establishment of this mark at telomeres, as well as that *Suv4-20dn* MEFs telomeric chromatin is more open than in *wt* cells. To test whether this change in chromatin structure affects the efficiency of reprogramming of telomeric chromatin in *Suv4-20dn* cells, we performed chromatin immunoprecipitation (ChIP) in *wt* and *Suv4-20dn* iPS cells (three or four independent iPS cells clones per genotype). Immunoprecipitated DNA was detected by Dot-Blot with a telomeric probe or a pericentric probe as a control.. As expected, *Suv4-20dn* iPS cells showed very low levels of H4K20 trimethylation at the telomeric and pericentric chromatin (Fig. 2 A–B) when compared to *wt* iPS.We show that *Suv4-20dn* iPS cells present similar density of H3K9me3 mark and TRF1 protein at telomeres (Fig. 2A), confirming a proper reprogramming of heterochromatin at telomeres in these cells.

### Telomeres elongate to a greater extent in Suv4-20dn iPS cells

**Figure 3:**
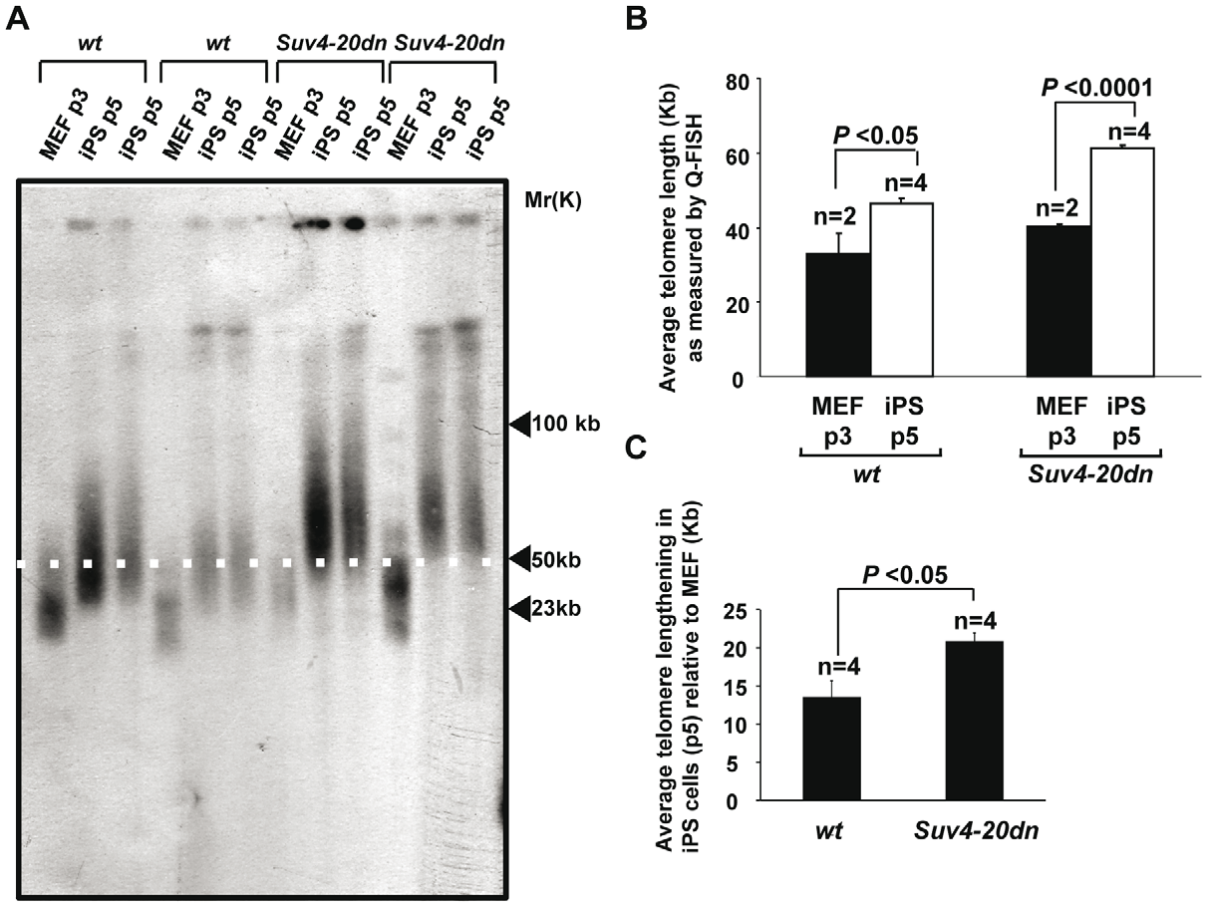
Enhanced telomere elongation in Suv4-20dn iPS cells. **a.** TRF results obtained with MEF (passage 3) derived from two embryos of each of the wild-type and *Suv4-20dn* genotypes and two independent iPS cells clones (passage 5) obtained from each MEF (a total of four independent iPS cells clones per genotype). Note the longer telomeres in *Suv4-20dn* MEF compared to *wt* MEF. Also note the telomere elongation in *wt* iPS cells when compared to the parental MEF, which is exacerbated in *Suv4-20dn* iPS cells. A white line is shown to facilitate the comparison of TRF sizes between different samples. **b.** Quantification of average telomere length (kilobases) as determined by Q-FISH on metaphases. n indicates number of independent MEF cultures or iPS cells colonies. Error bars correspond to standard error. Indicated statistics were performed using a Student's t-test. **c.** Average telomere lengthening (kilobases) in *wt* and *Suv4-20dn* iPS cells independent clones (passage 5) compared to their corresponding parental MEF (passage 3), as determined by Q-FISH on metaphases. Note the higher net telomere elongation in *Suv4-20dn* iPS cells, indicating an enhanced telomere lengthening in these cells. n indicates number of independent MEF cultures or iPS cells colonies. Error bars correspond to standard error. Indicated statistics were performed using a Student's t-test.

As part of the reprogramming process, telomeres elongate, and thus “rejuvenate”, in a telomerase-dependent manner, to finally reach hyper-long telomeres characteristic of ES cells [15]. It is thought that the remodeling of telomeric chromatin during reprogramming [15] could be required to allow telomerase access to the telomere and posterior telomere lengthening. Since telomeric chromatin in *Suv4-20dn* MEFs presents a lower density of the repressive H4K20me3 heterochromatic mark and a more open structure than that of *wt* cells, we set to determine whether this epigenetic difference could affect the efficiency of telomere elongation during reprogramming. To this aim, we measured telomere length of *wt* and *Suv4-20dn* iPS cells (four independent iPS cells clones per genotype, passage 5) and compared them with that of their corresponding parental MEFs. First, we measured the length of TTAGGG repeats using Southern blot terminal restriction fragment (TRF) analysis. In agreement with previous findings [27], abrogation of the repressive H4K20me3 chromatin mark at telomeres led to initially slightly longer telomeres in parental *Suv4-20dn* MEFs compared to *wt* MEFs (Fig. 3A). As expected, nuclear reprogramming resulted in net telomere elongation both in *wt* and *Suv4-20dn* iPS cells compared to their corresponding parental MEFs (Fig. 3A), indicating that the H4K20me3 mark is not required for efficient telomere elongation by telomerase. Interestingly, the relative increase in telomere length associated to nuclear reprogramming was greater in *Suv4-20dn* iPS cell clones compared to *wt* iPS clones, both at passage 5 post clone isolation, suggesting a more proficient telomere elongation in these cells. To confirm these data, we performed a quantitative FISH (Q-FISH) on metaphases using a telomere-specific probe. The results confirmed that initial average telomere length in *Suv4-20dn* MEFs (40.3 Kb) was higher than in wt MEFs (33.0 Kb) (Fig. 3B and Fig. S2) and that both *wt* (46.5 Kb) and *Suv4-20dn* (61.8 Kb) iPS cells at passage 5 show longer telomeres than their corresponding parental MEF (Fig. 3B and Fig. S2). Interestingly, when net telomere elongation in iPS cells was calculated (Fig. 3C), we observed a significantly higher increase in telomere length in *Suv4-20dn* iPS cells (20.8 Kb) than in *wt* iPS cells (13.5 Kb) when compared to their parental MEFs. These results indicate that telomeres elongate to a greater extent during reprogramming of *Suv4-20dn* cells when compared to *wt* controls.

### *Suv4-20dn* iPS cells produce more TERRA

Telomeres are actively transcribed in mammalian cells generating long, non-coding RNAs known as TelRNAs or TERRAs [38], [39], that are bound by RNA-binding proteins [40], [41]. They remain associated to the telomeric chromatin, where they have been proposed to act as negative regulators of telomere length [38]. TERRA levels are positively correlated with telomere length [38], suggesting that individual telomeres can produce different amounts of transcripts according to their length. We have previously described that, in agreement with their longer telomeres, TERRA levels are efficiently increased in iPS compared to the MEFs [15].

**Figure 4:**
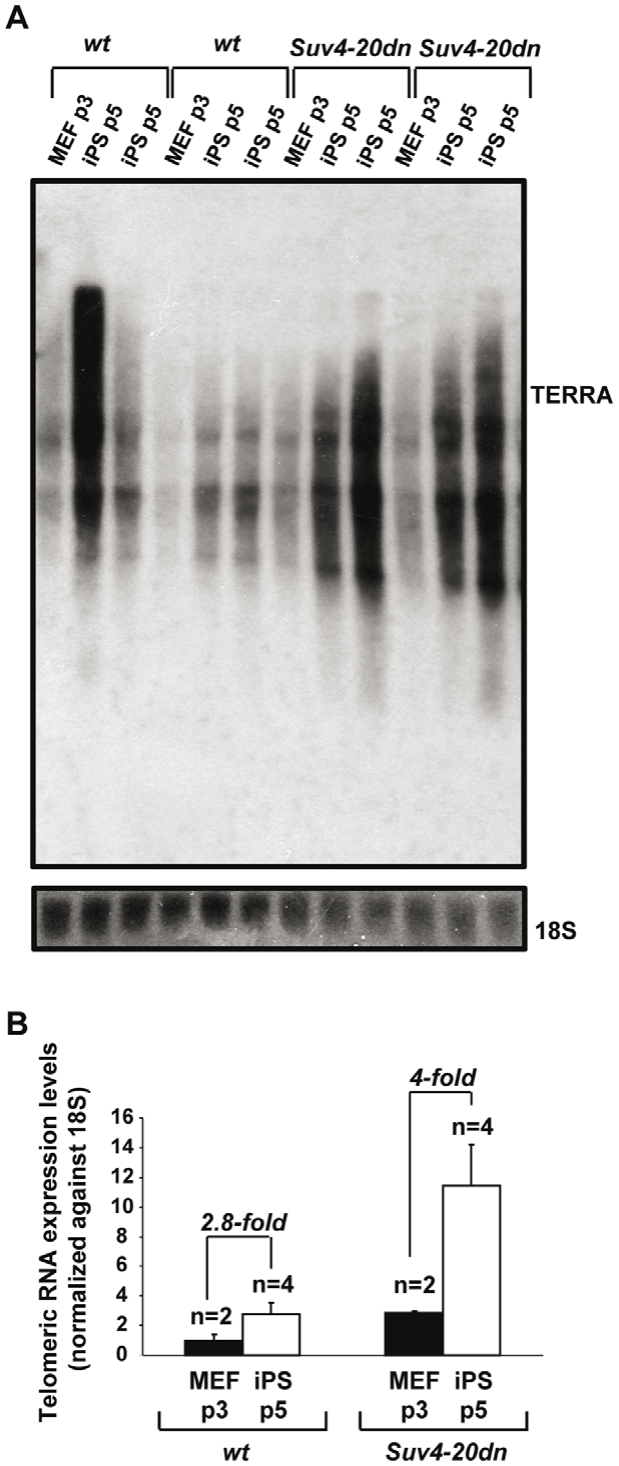
Increased levels of TERRA in *Suv4-20dn* iPS cells. **a.** Expression of TERRA in the indicated cells types as measured by Northern blot hybridization with a telomeric probe. A probe for the 18S ribosomal subunit was used as a loading control. **b.** Quantification of TERRA levels by Northern-blot shown in **a.** TERRA values were normalized against the signal for the 18S subunit to correct for differences in loading. Fold changes of iPS cells (passage 5) clones TERRA expression relative to their parental MEF (passage 3) are indicated. Note the higher increase in TERRA expression in *Suv4-20dn* iPS cells. n indicates number of independent MEF cultures or iPS cells colonies. Mean and standard error are shown.

 To assess the impact of loss of the H4K20m3 heterochromatic mark on TERRA expression on reprogrammed cells, we measured TERRA levels by northern blot analysis in *wt* and *Suv4-20dn* iPS cells (four independent iPS cells clones per genotype) and their corresponding parental MEFs. As previously described [38], and in agreement with their more relaxed telomeric chromatin, *Suv4-20dn* MEFs showed higher levels of TERRA than *wt* MEFs (Fig. 4 A and B). Interestingly, the fold increase in TERRA levels in *Suv4-20dn* iPS cells when compared to their parental MEFs (4 fold increase), was higher than in the case of *wt* cells (2.8 fold increase) (Fig. 4 A and B), in accordance to their enhanced telomere lengthening during reprogramming (Fig. 3 C). These results also support the notion that abundance of TelRNAs is positively correlated with telomere length.

### Chromosomal aberrations in Suv4-20dn iPS cells

**Figure 5:**
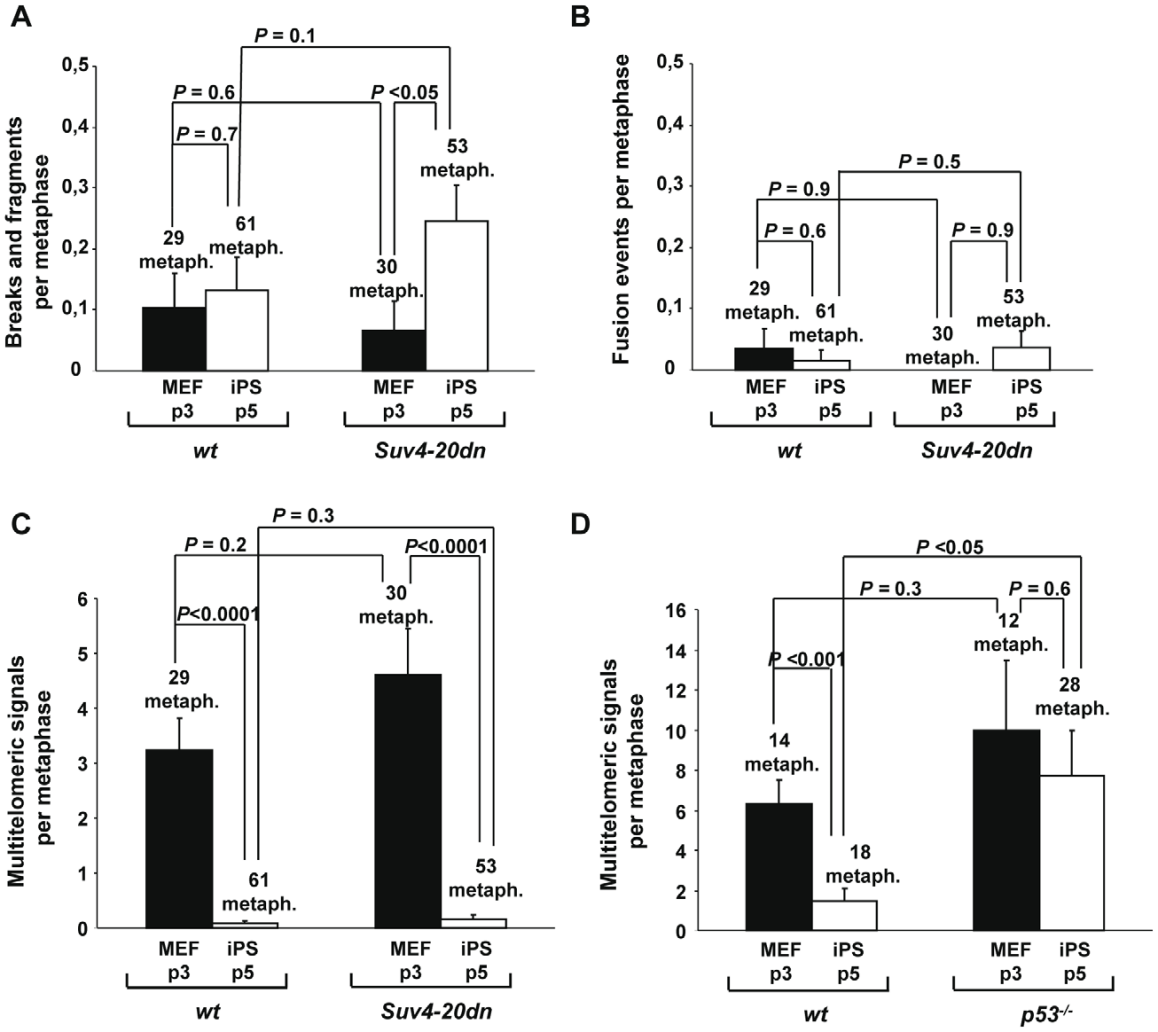
Abrogation of Suv4-20h enzymes does not increase telomeric aberrations in *Suv4-20dn* iPS cells. Frequency of (**a**) chromosomal breaks and fragments, (**b**) end-to-end fusions and (**c**) multitelomeric signals, in *wt* and *Suv4-20dn* MEF (passage 3) and iPS cells (passage 5). (**d**) Frequency of multitelomeric signals in *wt* and *p53^−/−^* MEF (passage 3) and iPS cells (passage 5). The number of metaphases analyzed in each case is indicated. Mean and standard error are shown. Indicated statistics were performed using a Student's t-test.

We previously showed that decreased abundance of H4K20me3 at the telomeres of *Suv4-20dn* MEFs does not lead to increased chromosomal aberrations involving telomeric repeats [27], suggesting that longer telomeres in *Suv4-20dn* cells remain fully capped. However, *Suv4-20dn* MEFs are more prone to chromosomal breaks and gaps as the result ionizing irradiation, suggesting an increased sensitivity of these cells to DNA damage [32]. Here, we set to address whether *Suv4-20dn* iPS cells were genetically stable or instead showed increased chromosomal aberrations. To assess this question, we performed fluorescent *in situ* hybridization (FISH) staining in metaphases using a telomeric probe, and analyze for the presence of chromosomal and telomeric aberrations. As shown in Fig. 5 A, *Suv4-20dn* iPS cells show an increased frequency of chromosomal fragments and breaks when compared to their parental differentiated MEFs, suggesting that reprogramming of the chromatin in cells lacking the H4K20me3 mark generates a conformation more fragile and prone to chromosomal breakage. In contrast, *Suv4-20dn* iPS cells show similarly low frequency of end-to-end fusion events (Fig. 5 B) and multitelomeric signals (Fig. 5 C) than *wt* iPS cells, indicating that reprogramming of telomeric chromatin in the absence of H4K20me3 mark does not alter the ability to protect the chromosome ends. Interestingly, our results show that both *wt* and *Suv4-20dn* iPS cells exhibit a significantly lower frequency of multitelomeric signals (MTSs) than their corresponding parental MEF, suggesting the existence of a selective mechanism that prevents the reprogramming of cells that carry this kind of telomere aberration, that results from increased telomere fragility and is increased under conditions of replication stress [37], [42], [43], [44]. It has been described that tumor suppressor p53 is critical in preventing the generation of human and mouse pluripotent cells from suboptimal parental cells carrying DNA damage, such as critically short telomeres or exogenously inflicted damage [45]. We asked whether p53 would also be involved in the inhibition of reprogramming of cells presenting MTSs. To address this question, we measured the frequency of MTSs in *wt* and *p53^−/−^* iPS cells and compared them with their corresponding parental MEF. As shown in Fig. 5 D, *p53^−/−^* iPS cells show frequencies of MTSs similar to their parental MEF, indicating a role of p53 in preventing reprogramming of cells with fragile telomeres.

### Teratomas derived from *Suv4-20dn* iPS cells grow faster and have longer telomeres

**Figure 6:**
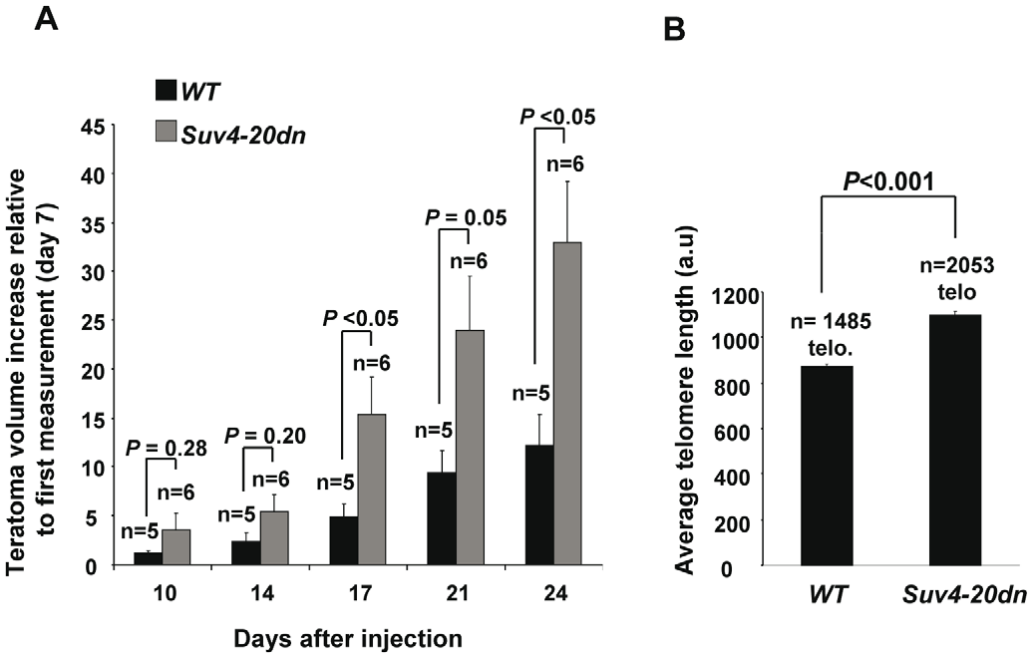
*Suv4-20dn* iPS cells-induced teratomas grow faster than the wt and have longer telomeres. **a.** Teratoma growth in mice (nu/nu) subcutaneously injected with 1×10^6^ cells of each *wt* and *Suv4-20dn* iPS cells. Teratoma growth rate was calculated by measuring their volume along several days after iPS cell injection and expressed as net volume increased relative to the first measurement. iPS cells were injected at passage 5. Note that teratomas derived from *Suv4-20dn* iPS cells present a higher growth rate that those derived from *wt* iPS cells. n indicates number of teratomas measured from each genotype. Mean and standard error are shown. Indicated statistics were performed using a Student's t-test. **b.** Quantification of average telomere length (a.u.) as determined by Q-FISH on *wt* and *Suv4-20dn* teratoma sections. Note the longer telomere length in *Suv4-20dn* teratomas compared with *wt* controls. n indicates number of telomeres analyzed for each genotype. Error bars correspond to standard error. Indicated statistics were performed using a Student's t-test.

The generation of tumor cells involves a series of epigenetic changes, such as global decrease in DNA methylation, H4K20 trimethylation and H4K16 acetylation [46]. Progressive loss of H4K20me3 and decreased levels of Suv4-20h2 have been associated with several cancers [47], [48], [49], [50], suggesting that abnormal regulation of H4K20me3 could be an important factor in oncogenesis or cancer progression. To further address the role of H4K20me3 and Suv4-20h enzymes in oncogenesis, we characterized the teratomas derived from injection of *wt* and *Suv4-20dn* iPS cells into immunodeficient mice. First, we calculated the growth rate of the teratomas by measuring their volume along several days after injection. As shown in Fig. 6 A, teratomas derived from injection of *Suv4-20dn* iPS cells grow significantly faster than those derived from *wt* iPS cells, indicating that loss of Suv4-20 enzymes and H4K20me3 mark provides a growth advantage to the tumor cells. We next addressed whether the differences in telomere length observed between the *wt* and *Suv4-20dn* iPS cells were reflected in the corresponding teratomas. To this aim, we performed telomere quantitative FISH (Q-FISH) analysis on teratoma sections. The results indicate (Fig. 6 B) that average telomere length in *Suv4-20dn* teratomas is higher than in the *wt* controls. The presence of longer telomeres associated with a faster teratoma growth rate supports a model were loss of H4K20me3 mark in *Suv4-20dn* cells favors an enhanced elongation of telomeres during reprogramming, which, in turn, may confer a growth advantage and increased tumorigenesis potential to the tumor cells

## Discussion

Current evidence suggests that chromatin remodeling proteins and reagents that induce a less compacted chromatin conformation also facilitate the reprogramming process [8], [10], [11], [12], [13], in accordance with the notion that elimination of silencing epigenetic marks is a key step in the generation of iPS cells. We previously showed that abrogation of Suv4-20h enzymes results in global loss of H4K20 di- and trimethylation heterochromatic marks and a global change to H4K20 monomethylation [27], [31], [32]. Here, we set to address the impact of this profound epigenetic change in nuclear reprogramming by generating *Suv4-20dn* iPS cells. Interestingly, we did not observe higher reprogramming efficiencies in *Suv4-20dn* cells compared to wild-type cells. This is most likely due to the fact that *Suv4-20dn* MEFs show severe proliferation defects and enter crisis earlier than *wt* cells [32]. Since reprogramming requires multiple cell division to silence lineage-specific genes and activate embryonic markers [51], the proliferative defect in *Suv4-20dn* MEFs could reduce their reprogramming capacity. Thus, it is plausible that only a subset of the infected cells would be able to complete the number of cell divisions required to fulfill the reprogramming process, thus masking a possible reprogramming advantage conferred by the presence of an open chromatin in these cells. In any case, we show here that reprogramming of *Suv4-20dn* cells generates *bona fide* iPS cells that express stemness markers, contribute to mouse chimerism and induce teratomas that differentiate in tissues derived from the three germ layers.

Nuclear reprogramming induces a series of profound changes at the telomeres of parental differentiated cells, leading to “rejuvenated” telomeres more similar to those of ES cells. In particular, during iPS cell generation, and as part of a genome-wide opening of the chromatin in this process, heterochromatic mark H4K20me3 is drastically reduced at telomeric chromatin. In addition, telomerase expression is activated and a continuous telomerase-dependent telomere elongation occurs, until reaching ES cell telomere length [14], [15]. A current model proposes that remodeling of telomeric chromatin may be required to allow telomerase access to the end of the telomere and posterior telomere lengthening. In line with this, we observe here that the loss of H4K20me3 in *Suv4-20dn* MEFs correlates with an enhanced telomere elongation during reprogramming of these cells, suggesting that the “relaxed” chromatin could facilitate the access of telomerase to the telomere during iPS cell generation. These data support the idea that chromatin remodeling at telomeres is a prerequisite for telomerase-dependent telomere elongation during reprogramming.

Telomeres function to guard chromosomes against degradation, fusions, and rearrangements [16]. Telomere capping depends on telomere length, telomeric chromatin structure and the shelterin complex [25], [52]. Loss of this telomere protection function results in chromosomal instability, including end-to-end fusions and occurrence of multitelomeric signals [52]. The results described here show that *Suv4-20dn* iPS cells present similarly low frequency of end-to-end fusion events and multitelomeric signals than *wt* iPS cells, indicating that they maintain their protective telomere function in the absence of the H4K20me3 mark. Multitelomeric signals are aberrant structures whose frequency increases under conditions of replication stress, such as aphidicolin treatment [43]. As telomeres resemble fragile sites [44], increased frequencies of MTS are a direct indicator of telomere fragility resulting from replication fork stalling [43], [44]. In this study, we observed that the frequency of MTS was greatly reduced in iPS cells compared to MEF, indicating that reprogramming of cells suffering of replicative stress is abolished. Interestingly, we show here that tumor suppressor p53, which is critical in aborting the reprogramming of cells carrying DNA damage, such as critically short telomeres or exogenously inflicted DNA damage [45], is also involved in preventing the reprogramming of cells that present higher loads of MTS, confirming a general role of p53 as a “reprogramming barrier” that prevents the generation of iPS cells from sub-optimal parental cells. It has been described that the DNA damage response kinase ATR, which functions to protect and stabilize stalled replication forks at common genomic fragile sites, has an important role in suppressing telomere fragility and occurrence of MTS [43], [53]. Thus, a possibility exists that, during reprogramming, the presence of MTS in parental cells could lead to activation of ATR kinase, ultimately resulting in a p53-mediated apoptosis process that would eliminate these cells to prevent the generation of genetically unstable iPS cells, although data supporting this hypothesis is not available.

Interestingly, p53 is critical to control the spreading of damaged cells in both reprogramming and malignant transformation, suggesting the existence of common mechanisms between reprogramming and cancer development. There are other common features shared between iPS reprogramming and tumorigenesis. Thus, in both cases the normal differentiation processes are altered, global epigenetic changes are necessary and the activity of transcriptional regulators is required to generate a new gene expression program according to the change in cell fate [54]. Also, reprogrammed cells and cancer cells activate telomerase expression [55], [56], [57], [58]. Moreover, as mentioned, these multistep processes have to overcome the common safety mechanism of “quality control” exerted by p53, designed to avoid the processes and maintain the cell identity, and thus, abrogation of p53 favors the occurrence of reprogramming and tumor initiation [45], [59], [60], [61], [62], [63]. Thus, a possibility exists that tumor initiation would start by de-differentiation of specialized cells that lose p53 activity and are more prone to reprogram, and that reprogramming would play a role in the development of cancer. In this regard, research in the field of reprogramming is opening new roads in the study of the molecular mechanisms of tumor initiation.

In line with this, here we show that abrogation of Suv4-20h enzymes enhances telomere elongation during reprogramming and generates iPS cells with a faster teratoma growth rate, indicating that loss of H4K20me3 mark provides a growth advantage and increased tumorigenesis potential to the tumor cells. These results are in agreement with the fact that a strong reduction of H4K20me3 and decreased levels of Suv4-20h2 have been associated with several cancers [47], [48], [49], [50], which suggests a possible role of Suv4-20h enzymes as tumor supressors. These data suggest that abnormal regulation of H4K20me3 confers a higher tumorigenic potential to both reprogrammed and transformed cells and that Suv4-20h enzymes could play an important role in oncogenesis or cancer progression. Due to the emerging connections between the processes of oncogenesis and de-differentiation, a deeper knowledge of the molecular mechanisms of reprogramming may open new roads to the identification of new genes involved in cancer initiation.

## Supporting Information

Figure S1 **Differentiation of** ***wt*** **and** ***Suv4-20dn*** **teratomas.** **a.** Percentage of teratoma area negative for Oct4 (left) and Nanog (right) staining in teratomas of the indicated genotypes. Note that *wt* and *Suv4-20dn* teratomas show very similar levels of the pluripotency markers, indicating comparable levels of cell differentiation. n indicates number of teratomas analyzed for each genotype. Error bars correspond to standard error. Indicated statistics were performed using a Student's t-test. iPS cells were injected at passage 5. **b.** Representative images of Oct4 and Nanog staining in *wt* and *Suv4-20dn* teratomas. Scale bar, 200 µm.
(TIF)

Figure S2 **Telomere length distributions of** ***wt*** **and** ***Suv4-20dn*** **MEF and iPS cells.** Quantification and distribution of telomere length (kilobases) of *wt* and *Suv4-20dn* MEF (passage 3) and iPS cells clones (passage 5) as determined by Q-FISH on metaphases. Red arrows indicate the iPS cells clones derived from each parental MEF.
(TIF)

Table S1 **Generation of chimeras from** ***wt*** **and** ***Suv4-20dn*** **iPS cells clones.** All the iPS cells used for microinjection in B6-tyrC-2J blastocysts or aggregation in CD1 morulae expressed Nanog and Oct-4 (see Fig. 1).
(TIF)
