# Supplementary figures and images for: Suv4-20h Abrogation Enhances Telomere Elongation during Reprogramming and Confers a Higher Tumorigenic Potential to iPS Cells
Source: PLoS One. 2011 Oct 12;6(10):e25680. doi: 10.1371/journal.pone.0025680 (PMC3192133; doi:10.1371/journal.pone.0025680)

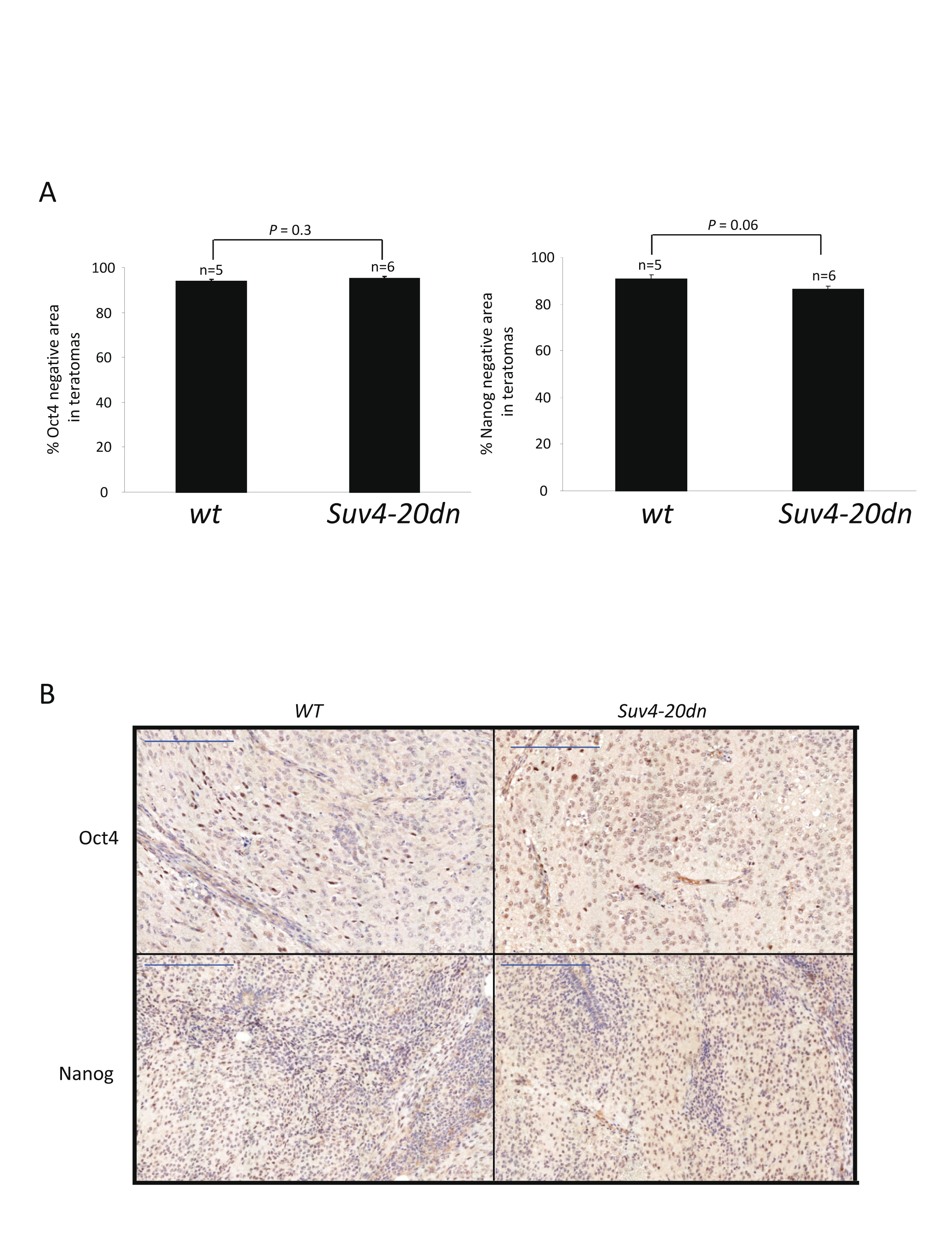

Supplement: Figure S1 — Differentiation of wt and Suv4-20dn teratomas. a. Percentage of teratoma area negative for Oct4 (left) and Nanog (right) staining in teratomas of the indicated genotypes. Note that wt and Suv4-20dn teratomas show very similar levels of the pluripotency markers, indicating comparable levels of cell differentiation. n indicates number of teratomas analyzed for each genotype. Error bars correspond to standard error. Indicated statistics were performed using a Student's t-test. iPS cells were injected at passage 5. b. Representative images of Oct4 and Nanog staining in wt and Suv4-20dn teratomas. Scale bar, 200 µm. (TIF) [file pone.0025680.s001.tif]

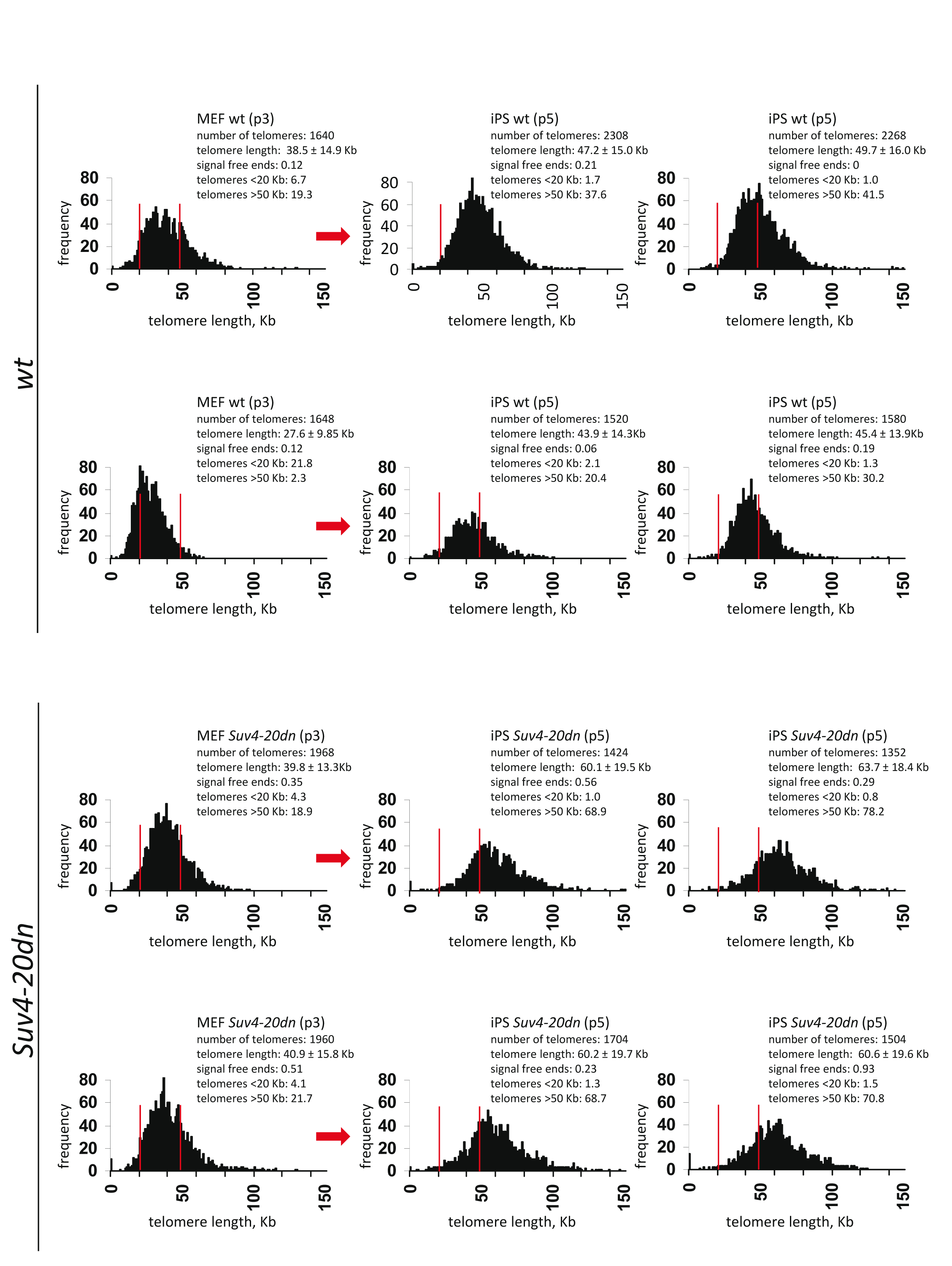

Supplement: Figure S2 — Telomere length distributions of wt and Suv4-20dn MEF and iPS cells. Quantification and distribution of telomere length (kilobases) of wt and Suv4-20dn MEF (passage 3) and iPS cells clones (passage 5) as determined by Q-FISH on metaphases. Red arrows indicate the iPS cells clones derived from each parental MEF. (TIF) [file pone.0025680.s002.tif]

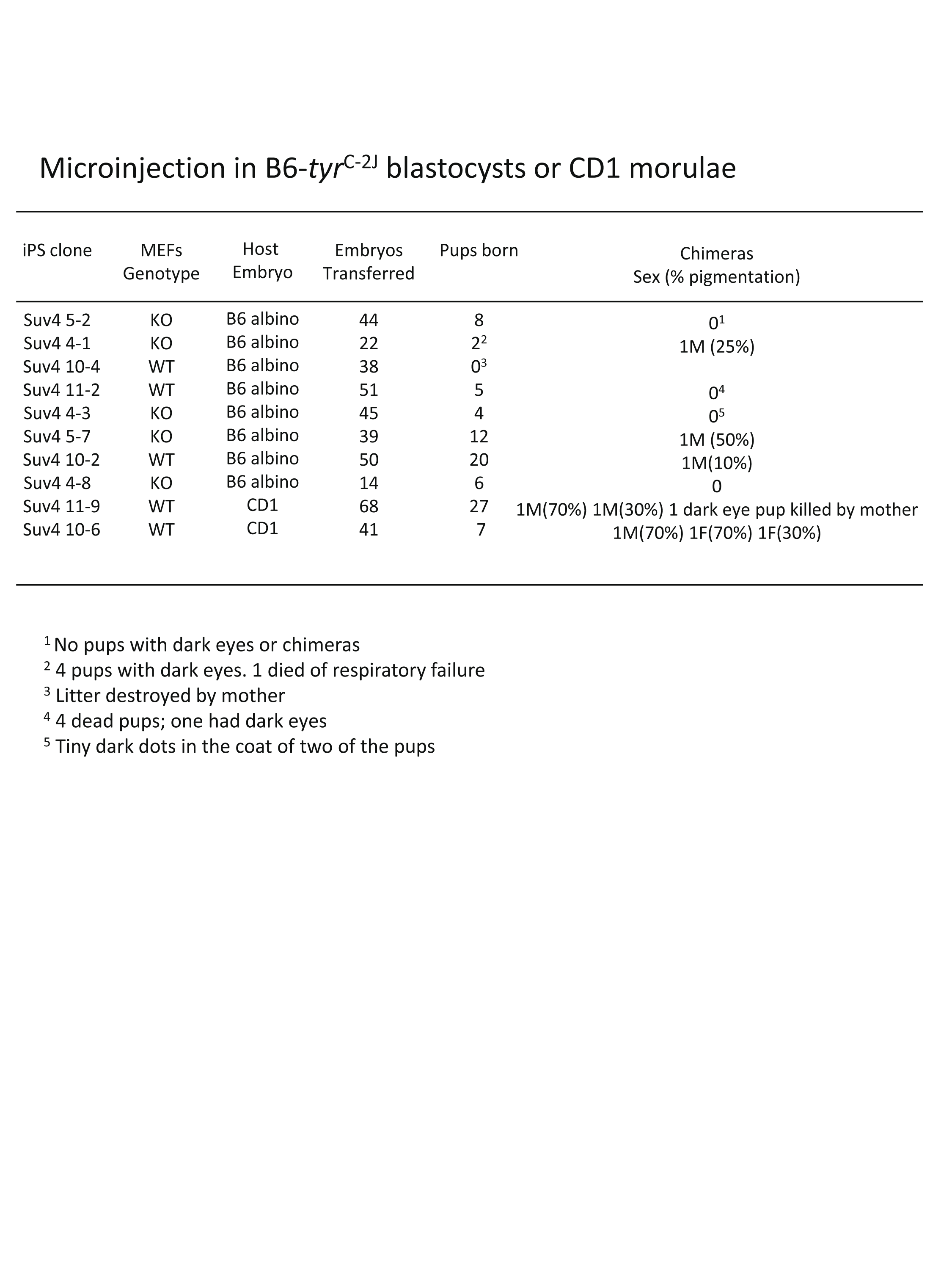

Supplement: Table S1 — Generation of chimeras from wt and Suv4-20dn iPS cells clones. All the iPS cells used for microinjection in B6-tyrC-2J blastocysts or aggregation in CD1 morulae expressed Nanog and Oct-4 (see Fig. 1). (TIF) [file pone.0025680.s003.tif]
